# Longer Health Resort Therapy Improves Outcomes in Long COVID: A Retrospective Study

**DOI:** 10.3390/medicina61091686

**Published:** 2025-09-17

**Authors:** Grzegorz Onik, Karolina Sieroń

**Affiliations:** School of Health Sciences in Katowice, Medical University of Silesia in Katowice, Department of Physical Medicine, Chair of Physiotherapy, 40-055 Katowice, Poland; ksieron@sum.edu.pl

**Keywords:** long COVID, balneotherapy, health resort treatment, therapy duration, adjuvant treatment, rehabilitation

## Abstract

*Background and Objectives*: The positive effect of health resort treatment on long COVID symptoms has been demonstrated. However, no previous study has considered therapy duration as a factor determining treatment effectiveness. Therefore, the objective of this study was to determine whether the duration of treatment predicts its effectiveness in individuals with long COVID. *Materials and Methods*: In this retrospective study, medical records of 119 individuals (68 women and 51 men; mean age 63.04 ± 8.61 years) undergoing health resort treatment for long COVID were analyzed. Participants were divided into two groups based on therapy duration: shorter (Group 1) and prolonged (Group 2). Dyspnea was assessed using the mMRC scale, physical performance with the SPPB, and functional status with the PCFS scale. Additionally, individuals rated symptom severity on 0–10 point scales. *Results*: Improvement in functional status was greater in individuals with a prolonged stay at the health resort (Group 1: 0.59 ± 0.66 points; Group 2: 1.41 ± 0.65 points; *p* < 0.001). Changes in the severity of most long COVID symptoms were significantly greater in patients who stayed longer. An extended stay at the health resort was associated with significant improvement in functional status (β = 0.033, *p* = 0.003) and in most long COVID symptoms, particularly sleep disorders (β = 0.112, *p* < 0.0001), memory disorders (β = 0.104, *p* < 0.0001), and headaches (β = 0.103, *p* < 0.0001). *Conclusions*: A prolonged stay in a sanatorium exerts a favorable effect on symptom severity in individuals with long COVID. Comprehensive health resort treatment of approximately four weeks is associated with improved functional status and alleviation of neuropsychiatric symptoms. Nevertheless, given the retrospective design of the present study, prospective research is required to validate these findings.

## 1. Introduction

It is estimated that 50–80% of hospitalized and 10–30% of non-hospitalized COVID-19 convalescents experience persistent symptoms lasting at least three months. Globally, the number of individuals affected by long COVID exceeds 65 million and continues to rise [1,2,3]. Long COVID poses both a significant public health challenge and an economic burden [4]. Affected individuals may present with a wide array of symptoms resulting from damage to multiple organ systems. Most commonly, convalescents report cardiorespiratory symptoms (e.g., dyspnea, reduced exercise capacity, fatigue, and cough) and neuropsychiatric symptoms (e.g., sleep disorders, cognitive dysfunction, headaches, and dysautonomia). Although the cardiovascular, respiratory, and nervous systems are most frequently affected, long COVID can also impact the gastrointestinal, reproductive, immune, and urinary systems [2]. Consequently, long COVID substantially diminishes patients’ quality of life [5,6]. In addition to its health implications, long COVID imposes considerable economic costs, including reduced work capacity, decreased productivity due to presenteeism, and a projected long-term decline in workforce participation [7]. To address these challenges, a range of interventions has been implemented, encompassing both pharmacological and non-pharmacological strategies [1]. These include rehabilitation [8], balanced diet and nutrition [9,10], psychological support [11,12], complementary medicine practices [13,14,15], and comprehensive health resort therapies [16,17,18,19,20,21].

Health resort treatment is an organized form of healthcare that utilizes natural therapeutic resources and climatic interventions for the treatment, rehabilitation, and prevention of various medical conditions [22,23]. Beyond therapy and rehabilitation, its goals also include health promotion and disease prevention, and this is achieved through a multidisciplinary approach incorporating balneological therapies, physical medicine modalities, structured exercise programs, psychological interventions, and health education [24,25,26,27]. Treatment is typically recommended for a minimum of ten days, with most programs lasting two to three weeks [28,29,30,31], and therapeutic effects have been shown to increase after 14 days, although shorter interventions may also be beneficial [32,33]. Evidence indicates that health resort therapy alleviates long COVID–related cardiopulmonary and neuropsychiatric symptoms while improving overall patient functioning [16,17,18,19,20,21], with treatment durations in post-COVID individuals ranging from 10 to 28 days [16,17,18,34,35]. To date, no study has examined the relationship between length of stay in a health resort and treatment effectiveness, which constitutes the aim of the present study.

## 2. Materials and Methods

### 2.1. Qualification Criteria

Medical records of individuals aged 42–79 years who completed the treatment course (i.e., underwent both pre- and post-treatment examinations) were included in the analysis. The records contained information on medical history, including comorbidities; sociodemographic data; types of interventions administered during the treatment course; assessment of persistent COVID-19 symptoms; dyspnea evaluation using the mMRC scale; functional status assessed with the Post-COVID-19 Functional Status Scale (PCFS); and physical performance evaluated using the Short Physical Performance Battery (SPPB).

Exclusion criteria were defined prior to the medical records review. Patients with selected neuropsychiatric, cardiovascular, pulmonary, or rheumatic conditions were excluded (Table 1), as were those with a history of cancer, limb amputation, or Lyme disease. Additionally, inability to participate in general exercise sessions was considered an exclusion criterion.

### 2.2. Data Collection

Data for this retrospective study were collected in 2021 at the Gwarek Rehabilitation Hospital and Sanatorium in Goczałkowice-Zdrój (Poland) from patients undergoing comprehensive health resort treatment for long COVID. The facility functions within the public healthcare system under a contract with the National Health Fund, while also offering private services. Treatment costs were covered by the National Health Fund in accordance with Regulation No. 63/2021, which was issued by the President of the National Health Fund. Patients provided informed consent to participate in the treatment program during their stay at the health resort. Medical records of 239 patients were reviewed between March and May 2024. Prior to the analysis, approval for this study was obtained from the authorities of the Gwarek Rehabilitation Hospital and Sanatorium, and all data were anonymized. The Bioethical Committee of the Medical University of Silesia in Katowice waived the requirement for formal ethical approval due to the retrospective nature of the study (decision number: BNW/NWN/0052/KB/238/23).

### 2.3. Individuals

Due to the exclusion criteria, the medical records of 89 individuals were withdrawn from this study. Of the remaining records, 31 were identified as incomplete and were therefore excluded to avoid compromising the statistical power of the analysis, as the missing data were determined to be completely at random. Ultimately, the medical records of 119 individuals (68 women and 51 men) who underwent health resort treatment for long COVID were included in the final analysis. For all patients, comprehensive treatment at the sanatorium was initiated within twelve months of recovery from COVID-19 (in accordance with legal requirements).

### 2.4. Treatment Regimen

Treatment in the health resort may last from 2 to 6 weeks, in accordance with Order No. 63/2021/DSOZ of the President of the National Health Fund. The treatment course was individually designed based on the symptoms reported by patients during the pre-therapy examination. This examination was conducted by an experienced medical doctor specializing in medical rehabilitation and/or balneology and physical medicine. The final examination was performed within 24 h prior to discharge upon completion of the treatment course. Comprehensive treatment in the health resort, as outlined in Order No. 63/2021/DSOZ of the President of the National Health Fund, included therapeutic exercises, physical medicine modalities, balneotherapy, health education and promotion, dietary consultations, and psychological support. According to legal regulations, each patient underwent a minimum of 96 procedures during the treatment period, with an average of at least 4 procedures per day. All long COVID patients participated in general developmental and respiratory exercises. Among physical medicine modalities, the most frequently administered treatments in long COVID patients were as follows: pneumatic massage (65.5% of individuals), low-level laser therapy (61.3% of individuals), and local cryotherapy (51.3% of individuals). Table 2 provides an overview of the physical medicine modalities and balneological interventions used in the treatment of long COVID patients in a health resort setting.

### 2.5. Methods of Assessment

The severity of long COVID cardiorespiratory and neuropsychiatric symptoms was assessed twice, during both the pre-treatment and post-treatment examinations conducted by the doctor, using a numeric rating scale. Prior to the evaluation, individuals were instructed that 0 points represented “no symptoms,” while 10 indicated “the most severe symptoms imaginable.” Moreover, patients were informed that this self-reported symptom severity was intended to reflect their actual status. Assessed cardiorespiratory symptoms included the following: dyspnea at rest, exercise-induced dyspnea, cough intensity, chest tightness, chest pain, sputum production, palpitations, and increased heart rate. In addition, dyspnea was also evaluated using the modified Medical Research Council (mMRC) scale. The neuropsychiatric symptoms evaluated included the following: concentration and memory disorders, headaches, dizziness, sleep disturbances, paresthesia, depression, and anxiety. In all inpatients, functional limitations resulting from COVID-19 were assessed using the Post-COVID-19 Functional Status Scale (PCFS), while physical performance was evaluated using the Short Physical Performance Battery (SPPB). Treatment effectiveness was calculated as the change (delta) in each variable, representing the difference between pre- and post-treatment values.

### 2.6. Statistical Analysis

Statistical analysis was performed using STATA 19 BE software. Qualitative variables are presented as frequencies and percentages, while quantitative variables are presented as means with standard deviations. The Chi-square (χ^2^) test was used to compare the qualitative (categorical) variables. Intragroup and intergroup comparisons were conducted with the Wilcoxon signed-rank test. To quantify the magnitude of the difference between groups, effect size was calculated using Cohen’s d. Linear regression, adjusted for age, sex, BMI, and baseline blood pressure, was used to assess the associations between the ∆ variables and treatment duration. Statistical significance was set at *p* < 0.05.

## 3. Results

Medical records of 119 individuals (68 women and 51 men), with a mean age of 63.04 years ± 8.61 years and who underwent health resort treatment due to long COVID, were analyzed. The patients’ mean body mass index was 30.48 kg/m^2^ ± 4.68 kg/m^2^. During the baseline examination, the mean systolic blood pressure was 139.73 mmHg ± 13.15 mmHg, and the mean diastolic blood pressure was 80.10 mmHg ± 7.64 mmHg. The average treatment duration was 24.29 ± 6.21 days, with a median of 21 days. Hypertension was the most commonly reported comorbidity among individuals with long COVID, affecting 38% of patients. Long COVID patients were divided into two groups based on treatment duration using the median of 21 days as the cut-off, which reflects the typical three-week course of health resort therapy. Patients with a treatment duration of 21 days or fewer were assigned to Group 1 (*n* = 73), whereas those who stayed longer were assigned to Group 2 (*n* = 46). In both groups, women outnumbered men. In Group 1, women constituted 53%, while in Group 2, they made up 63% (*p* = 0.30). Group characteristics are summarized in Table 3.

**Figure 1:**
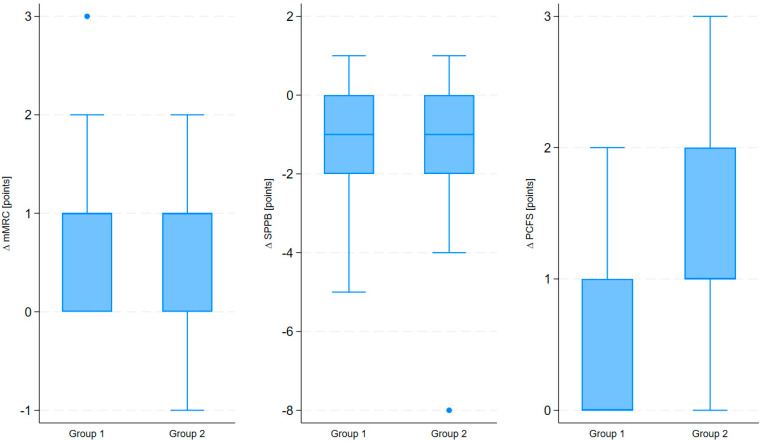
The changes in the mMRC, SPPB, and PCFS scores in the patient groups receiving health resort treatment.

In both groups, treatment was associated with significant improvements in mMRC, PCFS, and SPPB scores. In Group 1, the mean mMRC decreased by approximately 65% (*p* < 0.0001), PCFS by about 30% (*p* < 0.0001), and SPPB increased by approximately 9% (*p* < 0.0001). In Group 2, the mean mMRC decreased by approximately 58% (*p* < 0.0001), PCFS by about 56% (*p* < 0.0001), and SPPB increased by approximately 11% (*p* < 0.0001). Effect sizes (Cohen’s d) were as follows: mMRC at 0.11 (95% CI: −0.27; 0.47), PCFS at −1.25 (95% CI: −1.65; −0.85), and SPPB at 0.14 (95% CI: −0.23; 0.51). Figure 1 presents a comparison of the mean changes (∆) in the mMRC, PCFS, and SPPB scores between the two groups.

Both groups demonstrated significant improvement in the severity of most cardiopulmonary long COVID symptoms following comprehensive health resort treatment. In Group 1, the greatest improvements were observed in fatigue, malaise, exertional dyspnea, and cough (mean reduction ~2–2.5 points, *p* < 0.0001), whereas fast heart rate remained unchanged (*p* = 0.06). In Group 2, the most pronounced benefit was seen in fatigue and malaise (mean reduction > 3 points, *p* < 0.0001), while reductions in chest pain, palpitations, and fast heart rate did not reach statistical significance. Between-group comparisons revealed moderate effect sizes favoring Group 2 in fatigue, malaise, and resting dyspnea (−0.46 to −0.62), whereas improvement in exertional dyspnea was more pronounced in Group 1 (d = 0.52). For cough and cardiovascular symptoms, intergroup differences were negligible (Table 4).

Comprehensive health resort treatment led to significant improvement in neuropsychiatric long COVID symptoms in both groups of patients. In Group 1, the greatest reductions were observed in concentration disorders, memory disorders, sleep disturbances, and paresthesia (mean reduction ~1–1.6 points, *p* < 0.0001). In Group 2, more pronounced improvements were noted across all assessed symptoms. Fatigue in concentration and memory disorders showed a mean reduction of 2.43 points (*p* < 0.0001), while headaches and dizziness decreased by approximately 2 points (*p* < 0.0001). Between-group comparisons revealed moderate-to-large effect sizes favoring Group 2 for most symptoms, particularly for headaches (d = −1.12) and dizziness (d = −1.15). Improvements in concentration, memory, sleep, depression, and anxiety were moderate, whereas paresthesia showed a smaller intergroup effect (d = −0.32) (Table 5).

Linear regression analysis revealed that, among long COVID patients undergoing health resort treatment, the duration of therapy significantly predicted improvement in functional status, as assessed by the ∆ Post-COVID Functional Status (PCFS) scale. Each additional day of treatment was associated with an average 0.033-point improvement in functional status. However, no statistically significant associations were observed between treatment duration and changes in the mMRC or SPPB scores. Detailed results of the linear regression analysis are presented in Table 6.

Moreover, linear regression analysis revealed that treatment duration was significantly associated with improvements in selected cardiopulmonary long COVID symptoms, including ∆ fatigue, ∆ malaise, ∆ rest dyspnea, and ∆ sputum. The strongest association was observed for ∆ malaise, indicating that each additional day of treatment was associated with a 0.063-point improvement. However, the conducted analysis revealed that age was a significant independent predictor of change in fatigue (β = –0.041, *p* = 0.029) and malaise (β = –0.053, *p* = 0.008), indicating that older participants experienced less improvement in fatigue and malaise following treatment (Table 7).

**Figure 2:**
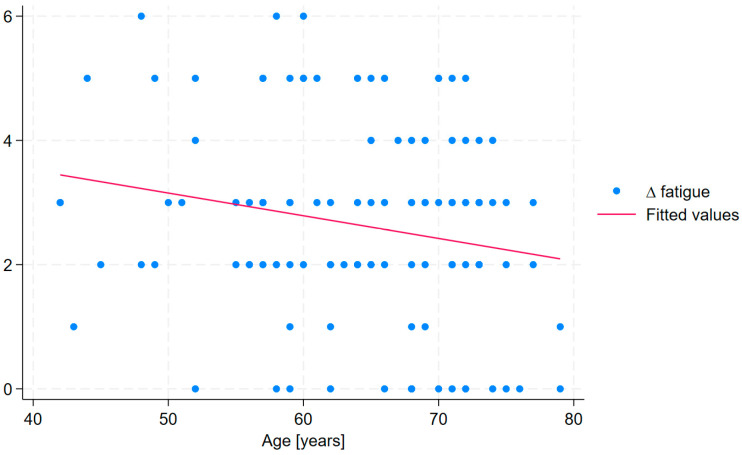
The association between age and change in fatigue (Δ fatigue) following treatment.

**Figure 3:**
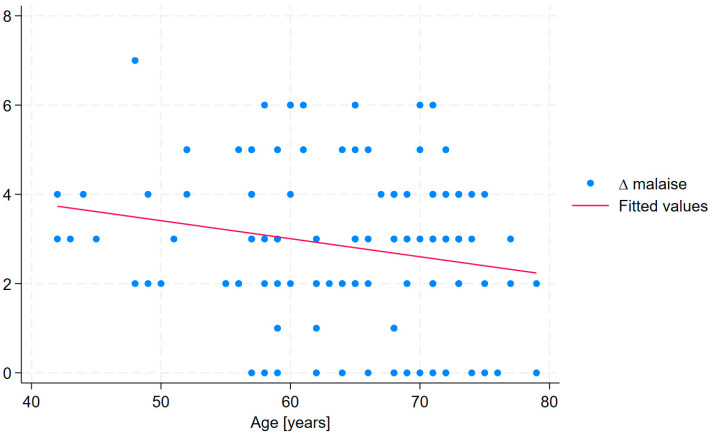
The association between age and change in malaise (Δ malaise) following treatment.

In the linear regression analysis, excluding the variable treatment duration rendered the overall model no longer statistically significant (R^2^ = 0.075, Adjusted R^2^ = 0.034, Prob > F = 0.117). However, age remained a significant predictor of change in fatigue, with older patients exhibiting a smaller increase in ∆ fatigue (β = −0.041, *p* = 0.031) (Figure 2). In the linear regression analysis of change in malaise (∆ malaise), when treatment duration was excluded, the overall model remained significant (R^2^ = 0.102, Adjusted R^2^ = 0.062, Prob > F = 0.033) and age continued to be a significant predictor (β = −0.054, *p* = 0.009) (Figure 3).

An association between treatment duration and improvement in neuropsychiatric long COVID symptoms was observed for most symptoms, with the exception of paresthesia (*p* = 0.21). The strongest relationship was found between ∆ sleep disorders and treatment duration, indicating that each additional day of treatment was associated with a 0.11-point improvement (Table 8).

## 4. Discussion

The key finding of our study is the higher effectiveness of comprehensive treatment in long COVID individuals who had a longer stay in the sanatorium. Prolonged treatment duration resulted in improvements in functional status, as assessed by the PCFS scale, as well as in most cardiopulmonary and neuropsychiatric symptoms. Moreover, we observed a significant association between treatment duration and both functional status improvement and the reduction in severity of certain long COVID symptoms. Until now, no study has considered the length of stay in a health resort as a predictor of treatment outcomes.

### 4.1. Functional Outcomes

Physical performance measured by the SPPB and dyspnea assessed using the mMRC scale did not differ between the groups of patients undergoing health resort treatment for long COVID. However, individuals who had a prolonged stay in the sanatorium showed greater improvement in functional status, as assessed by the PCFS scale. Ovejero et al. [36] reported that balneotherapy did not impact dyspnea (measured by the mMRC scale) or functional status (measured by the PCFS scale). In contrast, our study found that comprehensive health resort treatment had a favorable effect in individuals with long COVID who underwent a longer course of treatment. Although the study by Ovejero et al. [36] involved a four-week treatment duration, the frequency of administered modalities was lower compared to our study, which may explain the discrepancies in findings. Ponikowska et al. [35] postulated that comprehensive health resort treatment is more effective than treatment conducted outside the health resort due to the variety of therapeutic agents applied. In our study, individuals undergoing balneotherapy received treatment programs tailored to pre-treatment evaluations, ensuring a personalized and multifaceted therapeutic approach. Moreover, our findings are supported by those of Vancea et al. [37], who reported improvements in the functional capacity of individuals treated comprehensively (with at least five procedures per day) with rehabilitation and physical modalities for two weeks. A probable explanation for the better functional outcomes observed in Group 2 may be related to outdoor physical activity. Convalescent individuals could engage in walking exercises in the fresh air during their free time, which may have contributed to improvements in physical capacity and functional status [38].

### 4.2. Neuropsychiatric Long COVID Symptoms

Previous studies have demonstrated that balneotherapy alleviates fatigue in various patient populations [36,39,40,41,42]. Rapolienė et al. [39] reported that stationary balneotherapy significantly reduces fatigue, anxiety, and depression. Moderate effects have also been observed on sleep, memory, and concentration, whereas small effects have been noted on headaches and depression. Furthermore, the authors indicated that two-week interventions yield more favorable outcomes compared to one-week treatments. Consistent with our findings, individuals with a longer sanatorium stay demonstrated greater improvements in fatigue, concentration and memory disorders, sleep disturbances, depression, headaches, and anxiety compared to long COVID patients who underwent shorter treatment durations. The largest delta values were recorded for fatigue, indicating that this symptom was most positively influenced by the treatment. Lower delta values were observed for concentration disorders, memory problems, sleep disturbances, and headaches. The smallest changes among neuropsychiatric symptoms were found in depression and anxiety, which may reflect a trend similar to that described by Rapolienė et al. [39]. In contrast, in our study, neither short- nor long-term stays in the health resort had any effect on paresthesia, differing from the findings of Rapolienė et al. [39]. Bestaş et al. [42] conducted a four-week balneotherapy intervention and reported significant improvements in sleep quality. Similarly, our study demonstrated that long COVID patients with longer sanatorium stays experienced greater improvements in sleep disturbances, aligning with the results of Bestaş et al. [42]. Ovejero et al. [36] also reported enhanced sleep quality following balneotherapy administered three times per week over four weeks. In our study, each long COVID patient underwent at least four procedures daily. Although different instruments were employed to assess sleep quality across studies, which limits direct comparability, it may be assumed that the cumulative application of multiple procedures exerts a synergistic effect [32]. Despite the relatively low delta values for depression in both patient groups, some improvement was observed, with more favorable outcomes in Group 2. Moreover, García-López et al. [43] conducted a meta-analysis demonstrating that balneological treatment reduces depression, thereby supporting our findings.

### 4.3. Cardiopulmonary Long COVID Symptoms

The positive effects of various balneological agents on the cardiovascular system have already been reported [44,45,46,47]. Castelli et al. [25] suggested that balneotherapy positively influences microcirculation and stimulates adaptive responses in the autonomic nervous, endocrine, immune, and thermoregulatory systems. Furthermore, balneological interventions have been shown to benefit the respiratory system by improving expiration and reducing residual volume [48]. In our study, treatment with natural healing resources was combined with physical exercises, which have also been demonstrated to reduce dyspnea in individuals with long COVID [49]. Ponikowska et al. [35] reported improvement in dyspnea following at least a 12-day health resort stay, while Shchikota et al. [34] found that a 10-day treatment was beneficial for respiratory function. In line with these findings, our results demonstrated that individuals who underwent prolonged treatment in a sanatorium experienced significantly greater improvement in both rest and exertional dyspnea, as well as in cough and sputum production. Considering the recommendations by Maraver et al. [28], prolonged treatment should be advocated for individuals with long COVID.

### 4.4. Treatment Duration as a Predictor of Clinical Outcomes

Regression analysis revealed that extended treatment duration in a sanatorium improved the functional status of individuals with long COVID, with each additional day of treatment being associated with a 0.03-point increase on the PCFS scale. Bernard et al. [50] demonstrated that a three-week balneological treatment combined with exercise exerts a positive effect on physical functioning. In the present study, all participants engaged in exercise during their health resort stay. Prolonged treatment also facilitated increased recreational physical activity, which may have contributed to the observed outcomes [38]. Furthermore, treatment duration was significantly associated with improvements in fatigue and malaise, as well as in most neuropsychiatric symptoms. Dogaru et al. [51] postulated that the underlying mechanism of treatment effectiveness is not solely dependent on the cumulative impact of individual interventions, but also on the modulatory activity of the autonomic nervous system. The synergistic action of various therapeutic agents used in health resort treatment may induce adaptive responses [52]. Given the heterogeneous nature of long COVID and its multidirectional impact on human health [53], an extended length of stay appears justified, as it activates numerous biological processes—most notably, anti-inflammatory mechanisms that may alleviate symptoms [54]. However, in the present study, prolonged treatment was associated with improvements in only two cardiopulmonary symptoms. This finding may suggest that cardiac adaptation requires a longer timespan [55] and that extended treatment duration is not necessarily a predictor of cardiopulmonary symptom improvement.

Statistical analysis revealed that age was a significant predictor of changes in fatigue and malaise, thereby influencing treatment outcomes. Greater age was associated with reduced improvement in these symptoms. Age has previously been identified as a determinant of long COVID symptom severity [8,56,57], and current evidence indicates a high prevalence of fatigue among older adults [58,59]. Moreover, aging is physiologically linked to a marked decline in adaptive homeostatic responses [60], which may account for the observed association between age and persistent fatigue and malaise. These findings carry important clinical implications, suggesting that the duration of sanatorium stays should be extended for older individuals in order to stimulate adaptive responses. Although Latorre-Román et al. [61] reported that a 12-day course of balneotherapy was effective in elderly patients, prolonging the treatment duration may be advisable to potentiate the biological effects arising from the synergistic action of the administered therapies [58].

### 4.5. Study Limitations

Our study has several limitations. First, symptom severity was assessed subjectively using 0–10 point scales, which have not been formally validated. Nevertheless, subjective assessment may still reflect the perceived burden and impact of persistent COVID-19 symptoms on individuals. Second, participants in our study underwent personalized treatment protocols. Third, we did not assess the exact time interval between recovery from COVID-19 and admission to the health resort, as legal regulations only provided the timeframe from official clearance of infection to the start of treatment. This gap may have influenced symptom persistence and recovery trajectory. Fourthly, our study may have limited generalizability due to the exclusion of individuals with certain comorbidities. Nevertheless, we did include long COVID patients with comorbidities that have also been reported in population-based studies conducted by other researchers [62,63,64].

### 4.6. Future Directions

Future prospective studies are warranted to confirm the effectiveness of health resort treatment. To enhance the reliability of findings, a standardized treatment protocol should be implemented to ensure consistent outcome measurement and facilitate comparability across studies. This might be achieved by projecting studies that would consider employing validated instruments or objective measures to assess long COVID symptoms, such as the Pittsburgh Sleep Quality Index and the Fatigue Severity Scale. As physical activity was not evaluated in the present study, its assessment should be incorporated in future investigations, using tools such as the International Physical Activity Questionnaire (IPAQ) or pedometers. Finally, future studies should examine the economic burden associated with comprehensive health resort treatment for long COVID patients. Given the promising effectiveness observed in the current study, cost-effectiveness analyses comparing this approach with standard care could provide additional justification for integrating balneotherapy into long COVID rehabilitation strategies.

## 5. Conclusions

Individuals with long COVID who underwent prolonged stays in a health resort experienced better treatment outcomes. Extended duration of treatment was associated with greater functional improvement and significant alleviation of neuropsychiatric symptoms. Therefore, comprehensive health resort therapy should be considered, particularly for individuals with neurological complications resulting from COVID-19. A four-week treatment course may be optimal for long COVID patients. Nevertheless, prospective studies are needed to validate the results of our retrospective study.

## Figures and Tables

**Table 1:** Detailed exclusion criteria.

Neuropsychiatric Disorders	Cardiovascular Disorders	Respiratory Disorders	Rheumatic Disorders
Parkinson’s disease, multiple sclerosis, epilepsy, a history of stroke, depression, blindness	Coronary artery disease, heart failure, a history of myocardial infarction and/or endarterectomy, previous percutaneous coronary interventions and/or coronary artery bypass grafting, arrhythmias (atrioventricular or bundle branch blocks, and atrial fibrillation), pacemaker implantation, peripheral artery disease	Chronic obstructive pulmonary disease, emphysema, pneumoconiosis, asthma	Rheumatoid arthritis, ankylosing spondylitis

**Table 2:** The physical medicine modalities and balneological treatments applied during the health resort treatment of the long COVID patients.

Modality	*n*	% of Patients
Pneumatic massage	78	65.5%
Low-level laser therapy	73	61.3%
Local cryotherapy	61	51.3%
Infrared light therapy	47	39.4%
Whirlpool baths	47	39.5%
Magnetic fields	46	38.7%
Electrotherapy	39	32.8%
Pearl baths	27	22.7%
Peloids	15	12.6%
Classical massage	7	5.9%
Ultrasound therapy	7	5.9%
Lymphatic drainage	1	0.8%

**Table 3:** The demographic and clinical characteristics of the patient groups by treatment duration.

	Group 1 (*n* = 73)	Group 2 (*n* = 46)	*p* Value
Age [years]	63.66 ± 8.92	65.32 ± 8.08	0.31
Body mass [kg]	85.61 ± 14.24	84.69 ± 15.49	0.74
Height [m]	1.67 ± 0.08	1.67 ± 0.09	0.75
BMI [kg/m^2^]	30.59 ± 4.93	30.31 ± 4.29	0.75
Systolic blood pressure [mmHg]	140.61 ± 14.33	138.37 ± 11.12	0.37
Diastolic blood pressure [mmHg]	80.94 ± 8.02	78.80 ± 6.90	0.14
Treatment duration [days]	20.89 ± 0.54	29.67 ± 7.23	<0.0001
**Comorbidities**	**Group 1** (**% of individuals**)	**Group 2** (**% of individuals**)	***p*** **value**
Osteoarthritis	12%	35%	0.15
Diabetes mellitus + hypertension	14%	15%	
Hypertension	45%	26%	
Diabetes mellitus	1%	4%	
Diabetes mellitus + hypertension + hypothyroidism	1%	0%	
Diabetes mellitus + hypothyroidism	0%	2%	
Hypertension + hypothyroidism	6%	7%	
Hypertension + gout	4%	2%	
Hypothyroidism	6%	7%	
Benign prostatic hyperplasia	1%	0%	

**Table 4:** The effect of comprehensive health resort treatment on cardiovascular long COVID symptom severity across the patient groups, stratified by treatment duration.

		Fatigue [Points]	Malaise [Points]	Rest Dyspnea [Points]	Exertional Dyspnea [Points]	Cough [Points]
Group 1	Pre-treatment	4.4 ± 2.76	4.32 ± 2.87	0.76 ± 1.47	4.11 ± 2.83	0.83 ± 1.49
	Post-treatment	2.04 ± 1.43	1.86 ± 1.44	0.23 ± 0.54	1.71 ± 1.26	0.21 ± 0.49
	*p* value	<0.0001	<0.0001	<0.0001	<0.0001	<0.0001
	Mean ∆	2.35 ± 1.67	2.45 ± 1.75	0.53 ± 1.07	2.40 ± 1.94	0.63 ± 1.29
Group 2	Pre-treatment	5.39 ± 0.46	5.01 ± 1.57	1.72 ± 1.5	1.96 ± 1.96	1.17 ± 1.31
	Post-treatment	2.33 ± 1.48	1.59 ± 1.00	0.46 ± 0.72	0.48 ± 0.84	0.15 ± 0.42
	*p* value	<0.0001	<0.0001	<0.0001	<0.0001	<0.0001
	Mean ∆	3.07 ± 1.32	3.44 ± 1.50	1.26 ± 1.32	1.48 ± 1.50	1.02 ± 1.13
Cohen’s d		−0.46	−0.59	−0.62	0.52	−0.32
[95% CI]		−0.83; −0.08	−0.97; −0.22	−1.00; −0.24	0.14; 0.89	−0.69; 0.05
		**Sputum** [**points**]	**Chest** **tightness** [**points**]	**Chest pain** [**points**]	**Palpitations** [**points**]	**Fast heart rate** [**points**]
Group 1	Pre-treatment	0.37 ± 0.99	0.88 ± 1.74	0.29 ± 0.83	0.22 ± 0.71	0.22 ± 0.77
	Post-treatment	0.11 ± 0.4	0.18 ± 0.61	0.04 ± 0.2	0.06 ± 0.28	0.06 ± 0.23
	*p* value	0.0009	<0.0001	0.004	0.02	0.06
	Mean ∆	0.26 ± 0.69	0.69 ± 1.52	0.25 ± 0.70	0.16 ± 0.55	0.16 ± 0.73
Group 2	Pre-treatment	1.17 ± 1.22	0.52 ± 0.94	0.44 ± 1.17	0.24 ± 0.87	0.28 ± 0.91
	Post-treatment	0.17 ± 0.53	0.24 ± 0.6	0.12 ± 0.4	0.11 ± 0.48	0.13 ± 0.54
	*p* value	0.001	0.03	0.08	0.28	0.22
	Mean ∆	1.00 ± 1.01	0.28 ± 0.81	0.30 ± 1.09	0.13 ± 0.62	0.15 ± 0.67
Cohen’s d		−0.89	0.32	−0.07	0.06	0.02
[95% CI]		−1.28; −0.51	−0.05; 0.69	−0.44; 0.30	−0.31; 0.43	−0.35; 0.39

**Table 5:** The effect of comprehensive health resort treatment on neuropsychiatric long COVID symptom severity across the patient groups, stratified by treatment duration.

		Concentration Disorders [Points]	Memory Disorders [Points]	Headaches [Points]	Dizziness [Points]
Group 1	Pre-treatment	2.19 ± 1.39	2.56 ± 2.67	0.85 ± 1.76	0.74 ± 1.54
	Post-treatment	1.41 ± 1.38	1.41 ± 1.61	0.44 ± 1.01	0.36 ± 0.89
	*p* value	<0.0001	<0.0001	0.002	0.0005
	Mean ∆	1.08 ± 1.64	1.15 ± 1.69	0.41 ± 1.07	0.38 ± 0.95
Group 2	Pre-treatment	2.96 ± 2.26	2.89 ± 2.23	2.41 ± 2.06	2.35 ± 2.1
	Post-treatment	0.52 ± 0.81	0.46 ± 0.75	0.39 ± 0.68	0.33 ± 0.52
	*p* value	<0.0001	<0.0001	<0.0001	<0.0001
	Mean ∆	2.43 ± 2.17	2.43 ± 2.08	2.02 ± 1.89	2.02 ± 1.96
Cohen’s d		−0.73	−0.69	−1.12	−1.15
[95% CI]		−1.11; −0.35	−1.07; −0.31	−1.51; −0.72	−1.54; −0.75
		**Sleep disorders** [**points**]	**Paresthesia** [**points**]	**Depression** [**points**]	**Anxiety** [**points**]
Group 1	Pre-treatment	2.22 ± 2.31	2.37 ± 2.38	1.08 ± 1.91	0.89 ± 1.6
	Post-treatment	1.15 ± 1.43	0.75 ± 0.97	0.59 ± 1.31	0.39 ± 0.76
	*p* value	<0.0001	<0.0001	<0.0001	<0.0001
	Mean ∆	1.07 ± 1.58	1.61 ± 1.79	0.49 ± 1.02	0.51 ± 1.07
Group 2	Pre-treatment	2.98 ± 1.98	2.65 ± 1.98	2.09 ± 1.79	1.52 ± 1.57
	Post-treatment	0.39 ± 0.68	0.46 ± 0.72	0.30 ± 0.59	0.28 ± 0. 54
	*p* value	<0.0001	<0.0001	<0.0001	<0.0001
	Mean ∆	2.59 ± 2.01	2.20 ± 1.82	1.78 ± 1.65	1.24 ± 1.55
Cohen’s d		−0.86	−0.32	−1.00	−0.57
[95% CI]		−1.25; −0.48	−0.69; 0.05	−1.39; −0.6	−0.95; −0.19

**Table 6:** The predictive value of treatment duration for health resort outcomes in the long COVID patients: a regression model adjusted for age, BMI, baseline blood pressure, and sex.

		cons	β	SE	t	F(6, 110)	95% CI	R^2^	Adj. R^2^	*p* Value
Treatment duration	∆ PCFS	3.19	0.033	0.011	3.00	3.74	0.011; 0.054	0.169	0.124	0.003
	∆ SPPB	−2.28	−0.036	0.021	−1.74	2.85	−0.077; 0.005	0.135	0.088	0.085
	∆ mMRC	2.61	−0.011	0.011	−1.04	1.61	−0.033; 0.010	0.081	0.031	0.301

**Legend: cons**—intercept; **β**—unstandardized regression coefficient; **SE**—standard error of β; **CI**—confidence interval; and **adj. R**^2^—adjusted R-squared.

**Table 7:** The predictive value of health resort treatment duration for cardiopulmonary symptom improvement in the long COVID patients: a regression model adjusted for age, BMI, baseline blood pressure, and sex.

		cons	β	SE	t	F(6, 110)	95% CI	R^2^	Adj. R^2^	*p* Value
Treatment duration	∆ fatigue *	6.49	0.057	0.023	2.49	2.61	0.012; 0.102	0.125	0.077	0.014
	∆ malaise *	7.31	0.063	0.024	2.59	3.33	0.015; 0.112	0.154	0.108	0.011
	∆ rest dyspnea	1.46	0.037	0.018	2.01	1.01	0.0004; 0.016	0.052	0.0003	0.047
	∆ exertional dyspnea	2.46	−0.043	0.027	−1.61	2.48	−0.096; 0.01	0.119	0.071	0.110
	∆ cough	2.41	0.014	0.019	0.74	1.24	−0.023; 0.051	0.063	0.012	0.460
	∆ sputum	0.48	0.036	0.013	2.67	1.61	0.009; 0.062	0.081	0.031	0.009
	∆ chest tightness	4.13	−0.015	0.019	−0.75	1.77	−0.053; 0.024	0.088	0.038	0.458
	∆ chest pain	0.02	0.005	0.013	0.40	0.62	−0.021; 0.032	0.033	−0.02	0.689
	∆ palpitations	0.62	−0.001	0.009	−0.15	0.73	−0.019; 0.016	0.039	−0.014	0.879
	∆ fast heart rate	0.44	0.006	0.011	0.56	1.19	−0.015; 0.027	0.061	0.01	0.578

**Legend: cons**—intercept; **β**—unstandardized regression coefficient; **SE**—standard error of β; **CI**—confidence interval; **adj. R**^2^—adjusted R-squared; and *—age as a confounder.

**Table 8:** The predictive value of health resort treatment duration for neuropsychiatric symptom improvement in the long COVID patients: a regression model adjusted for age, BMI, baseline blood pressure, and sex.

		cons	β	SE	t	F(6, 110)	95% CI	R^2^	Adj. R^2^	*p* Value
Treatment duration	∆ concentration disorders	4.43	0.089	0.028	3.14	2.78	0.033; 0.145	0.132	0.084	0.002
	∆ memory disorders	3.02	0.104	0.027	3.79	4.17	0.05; 0.16	0.185	0.141	<0.0001
	∆ headaches	0.24	0.103	0.023	4.53	4.54	0.058; 0.148	0.198	0.155	0.0004
	∆ dizziness	−0.31	0.096	0.022	4.30	5.14	0.052; 0.141	0.219	0.176	<0.0001
	∆ sleep disorders	−1.88	0.112	0.027	4.37	3.91	0.064; 0.170	0.176	0.131	<0.0001
	∆ paresthesia	3.96	0.035	0.27	1.27	1.117	−0.2; 0.09	0.06	0.009	0.21
	∆ depression	1.05	0.072	0.021	3.48	2.88	0.031; 0.113	0.136	0.089	0.001
	∆ anxiety	2.56	0.047	0.019	2.44	2.03	0.009; 0.086	0.099	0.051	0.02

**Legend: cons**—intercept; **β**—unstandardized regression coefficient; **SE**—standard error of β; **CI**—confidence interval; and **adj. R**^2^—adjusted R-squared.

## Data Availability

The original contributions presented in this study are included in the article. Further inquiries can be directed to the corresponding authors.
